# A longitudinal study on the effects of COVID-19 pandemic on non-motor symptoms in Parkinson’s disease

**DOI:** 10.1007/s10072-022-06112-w

**Published:** 2022-05-10

**Authors:** Alfonsina D’Iorio, Chiara Baiano, Giovanna Maraucci, Carmine Vitale, Marianna Amboni, Gabriella Santangelo

**Affiliations:** 1 Department of Psychology, University of Campania “Luigi Vanvitelli”, Caserta, Italy; 2 Department of Motor Sciences and Wellness, University “Parthenope”, Naples, Italy; 3 Institute of Diagnosis and Care (IDC), Hermitage-Capodimonte, Naples, Italy; 4 Department of Medicine, Center for Neurodegenerative Diseases (CEMAND), University of Salerno, Salerno, Italy

**Keywords:** Parkinson’s disease, Non-motor symptoms, COVID-19, Social support

## Abstract

**Introduction:**

The COVID-19 pandemic led to psychological consequences on people’s mental health, representing a condition of increased vulnerability for the weakest sections of population, including elderly patients with Parkinson’s disease (PD).

This longitudinal study aimed at exploring the impact of the most frequent non-motor symptoms and their contribute on health-related quality of life of PD patients after the COVID-19 outbreak, in comparison with the pre-pandemic status.

**Methods:**

Forty-two non-demented PD patients underwent a first assessment between December 2018 and January 2020 (T0). Then, between March and May 2021 (T1), they were contacted again and asked to complete the second assessment. Levels of global functioning, several non-motor symptoms (i.e. depression, apathy, anxiety, anhedonia) and health-related quality of life were investigated.

**Results:**

Results of the the paired Wilcoxon signed-rank test showed that at T1, PD patients scored lower on the emotional subscale of the DAS, *Z* =  − 2.49; *p* = 0.013; *Cohen d*_*z*_ = 0.691. Higher scores of the TEPS total score, *Z* =  − 2.38; *p* = 0.025; *Cohen d*_*z*_ = 0.621, and LEDD, *Z* =  − 2.63; *p* = 0.008; *Cohen d*_*z*_ = 0.731, were also reported at T1.

**Conclusion:**

The present study suggested that self-isolation at home might lead to a reduction of apathy and anhedonia in PD patients due to the increase in social support provided by families during COVID-19 restrictions. This evidence brings out the need of a consistent and persistent social support which might be represented by caregivers or/and social assistive robotics.

## Introduction

The coronavirus disease 2019 (COVID-19) has greatly impacted people’s mental health, causing severe psychological consequences. Indeed, people experienced negative emotions (i.e. depressive symptoms, anxiety, emotional exhaustion, anger, trauma-related mental health disorders, insomnia) and negative cognitive assessment for self-protection such as fear of falling sick or dying and decreasing perception of wellbeing [1].

Particular groups appeared to be at higher risk for this kind of mental health impact, for instance the elderly, children, college students, homeless individuals and those in economic vulnerability and psychiatric patients [1]. Following the abovementioned findings, increased attention has been paid to the putative more severe consequences experienced by people with chronic neurologic diseases, such as patients with Parkinson’s disease (PD). In fact, some studies found that many PD patients are concerned about COVID-19 risk [2] and reported higher levels of stress and anxiety during the pandemic [3].

In addition, lockdown measures to mitigate the spread of COVID-19 deeply changed people’s daily lives in the short-term. According to Helmich and colleagues [4] successfully coping with such a sudden change requires cognitive operations depending on the normal functioning of dopaminergic structures and it is also well-known the link between impaired dopaminergic signalling and psychological post-traumatic stress [5].

Nigro-striatal dopamine depletion is the most relevant pathophysiological process in PD and, therefore, PD patients may be particularly vulnerable to negative psychological consequences following the COVID-19 pandemic and lockdown measures. Few cross-sectional surveys already provided insights about the greater vulnerability of PD patients to experience COVID-19 outbreak-related stress [2]. Another study [6] further explored whether pre-lockdown clinical features may be associated with the psychological impact of COVID-19 quarantine revealing that the psychological impact of a 40-day quarantine was associated with pre-lockdown levels of anxiety, treatment-related motor complications, quality of life and lockdown hours per day. However, until now, no longitudinal study systematically explored whether COVID-19 outbreak caused an aggravation of pre-existent non-motor symptoms and/or a deterioration in quality of life after the pandemic. Indeed, it is widely known that non-motor symptoms, such as apathy, anxiety and reduced functional autonomy are frequently reported in PD patient and can profoundly impact on their quality of life [7].

Taking into account the abovementioned reports, we aimed to explore the putative worsening of the most frequent non-motor symptoms in PD after the COVID-19 pandemic, by means of symptom-specific scales.

## Materials and methods

### Participants

For the present study, 68 PD outpatients were recruited from the Institute of Diagnosis and Care “Hermitage” of Naples (Italy). Participants underwent the first assessment between December 2018 and January 2020 (T0), before the COVID-19 outbreak. Then, between March and May 2021 (T1), they were contacted again and asked to complete the second assessment: 25 out of 68 participants did not give their consent for lack of interest or no time, so the final sample was of 42 PD patients. T1 neuropsychological assessment was conducted through telephone interview and demographic features (i.e. gender, age, years of schooling) were recorded. Clinical aspects (i.e. disease duration, Levodopa Equivalent Daily Dose, LEDD, severity of motor symptoms assessed by both part III of UPDRS and Hoehn and Yahr staging, H&Y) were recorded in person by neurologists during routine exams at the clinical facility. To be included in the study, each PD patient had to be without a clinical diagnosis of dementia or cognitive impairment (defined as total age-and education-adjusted score ≥ 15.5 at *Montreal Cognitive Assesment* (MoCA [8]) and with no clinical diagnosis of major concurrent neurological disorders. Furthermore, for each PD patient, severity of motor symptoms and psychiatric therapy must have been stable between T0 and T1 and they must not have undergone any kind of rehabilitation interventions (e.g. use of neuromodulation techniques such as Transcranial Direct Current Stimulation) from T0 to T1.

The research was conducted after PD patients provided their consent approved by the Local Ethics Committee and performed in accordance with the ethical standards laid down in the 1964 Declaration of Helsinki. Socio-demographic aspects, such as age and education level, were recorded. Moreover, before starting the second assessment at T1, each of participants was asked if they had contracted the COVID-19 virus.

### Measures

The Montreal Cognitive Assessment (MoCA [8]) assessing global functioning was administered at T0. Moreover, all PD patients completed self-report questionnaires assessing some of the most frequent non-motor symptoms both at T0 and at T1.

Anxiety was assessed by the Parkinson Anxiety Scale (PAS [9]), a self-report scale composed by 12-items rated on a 5-point Likert scale, with 0 representing “not or never” and 4 representing “severe or almost always”, divided into three subscales: persistent anxiety (5 items), episodic anxiety (4 items) and avoidance behaviour (3 items). The total score range is from 0 to 48, with higher scores indicating more severe anxiety (cut-off score ≥ 9).

Depression was assessed by the Beck Depression Inventory II (BDI-II [10]), a 21-item self-report inventory measuring the severity of depression. The score range is from 0 to 63, with higher scores indicating more severe depressive symptoms.

Apathy was assessed by the Dimensional Apathy Scale (DAS [11]), a self-report questionnaire composed by 24-items rated on a 4-point Likert scale, with 0 representing “almost always” and 4 representing “almost never”. The total score range is from 0 to 72, with higher score indicating more severe apathy.

The presence of anhedonia was assessed by the Temporal Experience of Pleasure Scale (TEPS [12]), a self-report scale evaluating two hedonic components: anticipatory pleasure (10 items) and consummatory pleasure (8 items). It consists of 18 items rated on a 6-point Likert scale, with 1 representing “very false” and 6 “very true”. The score range is from 18 to 108, with lower scores indicating the presence of anhedonia.

The Parkinson’s Disease Quality of Life Questionnaire (PDQ-8 [13]) was used to measure self-perceived health and health-related quality of life (HRQoL). It consists of 8-items that refers to how patients have experienced difficulties due to PD in the preceding month, rated on a 5-point Likert scale, with 0 representing “never” and 4 representing “always”. The total score ranges from 0 to 100, with lower scores indicated greater QoL levels.

### Statistical analysis

Preliminary descriptive analyses on demographic (i.e. gender, age, years of schooling), clinical (i.e. disease duration, Levodopa Equivalent Daily Dose, UPDRS, H&Y) and cognitive (MoCA) features at T0 were executed. The percentage of PD patients which contracted the COVID-19 virus at T1 in was also computed. Since there were several significant violations of assumption of normality (Shapiro–Wilk *p*-value < 0.05), non-parametric statistics were conducted. The paired Wilcoxon signed-rank test was conducted on clinical (i.e. LEDD, UPDRS-III, H&Y) and behavioural (i.e. PAS, BDI-II, DAS, TEPS, PDQ-8 scores and sub-scores when provided) features to investigate differences between T0 and T1. Effect size power analysis was calculated using Cohen’s d_Z_ measure. The critical alpha level for all analyses was set < 0.05. All the analyses were performed using the Statistical Package for Social Sciences (SPSS Inc, version 25).

## Results

Descriptive analyses on demographic (i.e. gender, age, years of schooling), clinical (i.e. disease duration, Levodopa Equivalent Daily Dose, UPDRS-III, H&Y) and cognitive (MoCA) features at T0 are reported in Table 1. The percentage of PD patients which contracted the COVID-19 virus at T1 in was 9.5% (4 out of 42).

**Table 1:** Descriptive statistics of demographic, clinical and cognitive features at T0 for the total sample (*N* = 42)

	Mean	SD	Minimum	Maximum
Age, years	65.24	8.29	44	82
Education, years	10.90	4.67	3	18
nM/nF	26/16	-	-	-
Disease duration, years	10.16	5.64	2	25
UPDRS-III	14.28	7.77	3	34
H&Y	2.2	0.64	1.5	4
LEDD	646.19	354.57	150	1465
MoCA total raw score	21.02	4.27	13	28
Visuospatial abilities	2.56	1.04	1	4
Executive functions	1.88	1.45	0	4
Language	4.60	1.44	1	6
Orientation	5.88	0.44	4	6
Attention	4.84	1.14	2	6
MoCA total adjusted score	21.91	3.14	15.5	27.6
PAS score	10.70	9.5	0	37
BDI-II score	8.1	7.06	0	27
DAS total score	22.55	9.32	6	56
DAS emotional	7.83	4.68	0	18
DAS executive	5.36	4.92	0	21
DAS behavioural/cognitive initiation	9.36	4.69	1	22
PDQ-8 total score	8.07	6.93	0	26
TEPS total score	68.77	14.83	36	90
TEPS anticipatory pleasure	34.15	10.22	13	49
TEPS consummatory pleasure	36.92	7.45	23	47

*SD*, standard deviation; *nM*, number of males; *nF*, number of females; *UPDRS-III*, Unified Parkinson’s Disease Rating Scale, part III; *H&Y*, Hoehn and Yahr staging; *LEDD*, Levodopa Equivalent Daily Dose; *MoCA*, Montreal cognitive assessment; *PAS*, Parkinson Anxiety Scale; *BDI-II*, Beck Depression Inventory II; *DAS*, Dimensional Apathy Scale; *PDQ-8*, The Parkinson’s Disease Quality of Life Questionnaire; *TEPS*, Temporal Experience of Pleasure Scale

Results of the paired Wilcoxon signed-rank test (Table 2) showed significant main effects of time of the assessment (T0 vs. T1) on the emotional subscale of the DAS, *Z* =  − 2.49; *p* = 0.013; *Cohen d*_*z*_ = 0.691, with higher scores at T0 compared to T1. A main effect of time of the assessment was also found on LEDD, *Z* =  − 2.63; *p* = 0.008; *Cohen d*_*z*_ = 0.731, and TEPS total score, *Z* =  − 2.38; *p* = 0.025; *Cohen d*_*z*_ = 0.621, with higher scores at T1 compared to T0. Results of the paired Wilcoxon signed-rank test did not show any other main effect on clinical and behavioural features (all *p* > 0.05).

**Table 2:** Results of the paired Wilcoxon test on clinical and behavioural features with time of the assessment as a between-subject factor

	T0	T1	*Z*	*p*	Cohen d_z_
LEDD	646.19 ± 354.57	848.4 ± 432.54	− 2.63	0.008	0.731
UPDRS-III	14.28 ± 7.77	13.52 ± 7.65	− 1.24	0.213	0.345
H&Y	2.2 ± 0.64	2.37 ± 0.69	0.00	1.00	0.000
PAS score	10.7 ± 9.5	8.10 ± 6.56	− 1.75	0.079	0.487
BDI-II score	8.10 ± 7.06	8.02 ± 4.47	− 0.85	0.392	0.237
DAS total score	22.55 ± 9.32	20 ± 8.2	− 1.68	0.093	0.466
DAS emotional	7.83 ± 4.68	5.64 ± 3.5	− 2.49	0.013	0.691
DAS executive	5.36 ± 4.92	5.88 ± 4.6	− 0.64	0.520	0.178
DAS behavioural/cognitive initiation	9.36 ± 4.69	8.64 ± 4.9	− 0.80	0.423	0.222
PDQ-8 total score	8.07 ± 6.93	6.88 ± 4.66	− 1.03	0.300	0.277
TEPS total score	68.77 ± 14.83	81.29 ± 6.74	− 2.23	0.025	0.621
TEPS anticipatory pleasure	34.15 ± 10.22	40.55 ± 4.57	− 1.22	0.221	0.340
TEPS consummatory pleasure	36.92 ± 7.45	40.76 ± 4.14	− 1.71	0.086	0.477

The values are expressed as mean ± standard deviation; *T0*, before the COVID-19 outbreak; *T1*, after the COVID-19 outbreak; *LEDD*, Levodopa Equivalent Daily Dose; *UPDRS-III*, Unified Parkinson’s Disease Rating Scale, part III; *H&Y*, Hoehn and Yahr staging; *PAS*, Parkinson Anxiety Scale; *BDI-II*, Beck Depression Inventory II; *DAS*, Dimensional Apathy Scale; *PDQ-8*, The Parkinson’s Disease Quality of Life Questionnaire; *TEPS*, Temporal Experience of Pleasure Scale

## Discussion

The main aim of the present study was to explore the impact of COVID-19 pandemic on the most frequent non-motor symptoms, such as depression, apathy, anxiety and anhedonia, in patients with PD by using symptom-specific scales. Overall, results showed a reduction of emotional apathy and anhedonia compared to the pre-COVID condition. These results seemed surprising since some recent studies suggested that PD patients may be more susceptible for negative psychological and psychosocial effects of the isolation and other restrictions due to pandemic [4]. A similar study [14] showed an impairment of non-motor symptoms in PD patients during the lockdown, in particular anxiety and cognition.

On the other hand, a study by HØrmann Thomsen et al. [15] found that PD patients experienced less sleep disturbances during the COVID-19 compared to pre-COVID-19 period. In addition, the authors revealed an improvement in HRQoL, despite increased anxiety. In the abovementioned study, participants indicated that the improvement in quality of life was due to not having usual social pressures, enjoying life being simple and slow and enjoying being at home.

A recent review [16] highlighted that also other studies found no significant worsening of mental health problems or only a minority of participants reporting worsening mental health symptoms.

Our results which, on the surface, might been positive should be explained by the fact that PD causes many people to withdraw from their social roles causing deficits in corresponding activities. Indeed, PD symptoms (e.g., motor symptoms, neuropsychiatric symptoms) negatively interfered with relationships [17]. Therefore, many PD patients already live in a state of social isolation and, paradoxically, social restrictions due to pandemic could have a less impact on this patient population. Moreover, for the purpose of our work, it is necessary to highlight that all patients included in the study were discharged from rehabilitative interventions before COVID-19 and they all lived at home with a caregiver.

Considering these issues, it is possible to hypothesize that PD patients experienced lower social pressure and benefited from greater social support from their caregiver and, as a consequence, they also experienced a reduction of emotional apathy and anhedonia.

As for clinical aspects, we observed an increase of LEDD from T0 to T1; this increase might be associated with the improvement of emotional apathy and anhedonia although we did not observed any association between the increase of LEDD and levels of apathy and anhedonia (data not shown) Moreover, nowadays, the efficacy of dopaminergic treatment on reduction of non-motor symptoms in PD is controversial and this issue deserves to be better investigated [18].

Before concluding, it should be noted that one possible limitation of our study is that our sample size was relatively small and not entirely representative of the PD population. Thus, our results do not allow for an advance in any conclusion regarding the impact of COVID-19 on PD in general. However, the findings of the present study point to the urgency of taking into account that in chronic illness, including in PD, symptoms impact the ability to fulfil social roles, causing social isolation and loneliness which are risk factors for increased health care costs and mortality [17]. The abovementioned evidence highlights the need of a consistent and persistent social support which might be represented by caregivers or/and social assistive robotics.
